# Comprehensive analysis of ALG3 in pan-cancer and validation of ALG3 as an onco-immunological biomarker in breast cancer

**DOI:** 10.18632/aging.205483

**Published:** 2024-02-07

**Authors:** Xiaolei Xue, Qiaoli Feng, Xi Hong, Zhousheng Lin, Yingrui Luo, Yingshi Li, Guangyu Yao, Nisha Wang, Lujia Chen

**Affiliations:** 1 Breast Center, Department of General Surgery, Nanfang Hospital, Southern Medical University, Guangzhou, Guangdong 510515, P.R. China; 2 Department of Pathology, Nanfang Hospital, Southern Medical University, Guangzhou, Guangdong 510515, P.R. China; 3 Department of Biochemistry and Molecular Biology, School of Basic Medical Sciences, Southern Medical University, Guangzhou, Guangdong 510515, P.R. China; 4 Basic Medical Academy, Cancer Research Institute, Southern Medical University, Guangzhou, Guangdong 510515, P.R. China

**Keywords:** ALG3, pan-cancer, breast cancer, TME, biomarker

## Abstract

ALG3 has significant modulatory function in the process of tumor development. Yet how ALG3 involves in the advancement of different malignancies isn’t fully understood. We performed a pan-cancer assessment on ALG3 utilizing datasets from The Cancer Genome Atlas (TCGA) and Genotype-Tissue Expression (GTEx) to examine its tumor-related roles across malignancies and its link to particular molecules and cells in the tumor microenvironment (TME). Furthermore, we focused on breast cancer to examine the influence of ALG3-mediated signaling pathways and intercellular interactions in the advancement of tumors. The biological effects of ALG3 were verified by breast cancer cells. Enhanced ALG3 expression was discovered to be substantially linked to patients' grim prognoses in a number of malignancies. Furthermore, the expression of ALG3 in the TME was linked to the infiltration of stromal and immune cells, and ALG3-related immune checkpoints, TMB, and MSI were also discovered. We also discovered that cancer patients having a high level of ALG3 exhibited a lower probability of benefiting from immunotherapy. Furthermore, our research found that KEGG enrichment, single-cell RNA and spatial sequencing analyses were effective in identifying key signaling pathways in ALG3-associated tumor growth. *In vitro*, knockdown of ALG3 could decrease the proliferation of breast cancer cells. In summary, our research offers a comprehensive insight into the advancement of tumors under the mediation of ALG3. ALG3 appears to be intimately associated with tumor development in the TME. ALG3 might be a viable treatment target for cancer therapy, particularly in the case of breast cancer.

## INTRODUCTION

Alpha-1,3-mannosyltransferase (ALG3) is situated in the Golgi apparatus and endoplasmic reticulum and is engaged in the production of early N-glycans [1]. In tumor cells, ALG3 is involved in the production of high-mannose type N-glycans [2]. It has been demonstrated that the progression of cancer is linked to the abnormal expression of multiple high-mannose type N-glycans [3]. ALG3 overexpression results in glycoprotein malfunction, which promotes tumor cell proliferation and invasion [4]. According to several reports, ALG3 is upregulated in squamous cell cervical cancer as well as esophageal squamous cell carcinoma and is responsible for inducing resistance to drugs in myeloid leukemia [5–7]. Under-glycosylation of glycosylated proteins and malfunctioning of glycoproteins are the consequences of ALG3 mutations [8]. Nonetheless, further investigation is warranted to shed light on the clinical value of ALG3 and its function in pan-cancer.

Recently, the advent of high-throughput sequencing-based cancer atlas programs in conjunction with omics technology has introduced new avenues of insight into the field of oncology research [9, 10]. Transcriptome innovation has long been utilized to comprehend the functions of the expression of genes in tumor cells, and recent de convolution network techniques enable tumor researchers to identify immune cells’ expression patterns from transcriptomic data and characterize their distributions [11, 12]. Furthermore, the massive quantity of data created by the unparalleled bioinformatics advancement may be utilized to deliver a comprehensive view of all identified genes and tumor types [13, 14]. This bio-informatic analysis, which uses several database systems to assess the expressions, prognoses, gene mutation patterns, and functionality of genes in distinct cancers, is known as pan-cancer analysis, and it may be employed to explore the functions and relationships of genes in distinct malignancies [15, 16]. Therefore, our research aims to investigate ALG3 by a pan-cancer investigation of the integrated multi-database.

In this research, we conducted a pan-cancer analysis to examine the link between ALG3 expression, patient prognosis, immunological milieu, microsatellite instability (MSI) immune checkpoint genes, tumor mutation burden (TMB), and immunological neoantigens in 33 cancers. In addition, we validated the biological function and explored the underlying mechanisms of ALG3 in breast cancer.

## RESULTS

### Expression levels and prognostic analysis of ALG3 in pan-cancer

**Figure 1:**
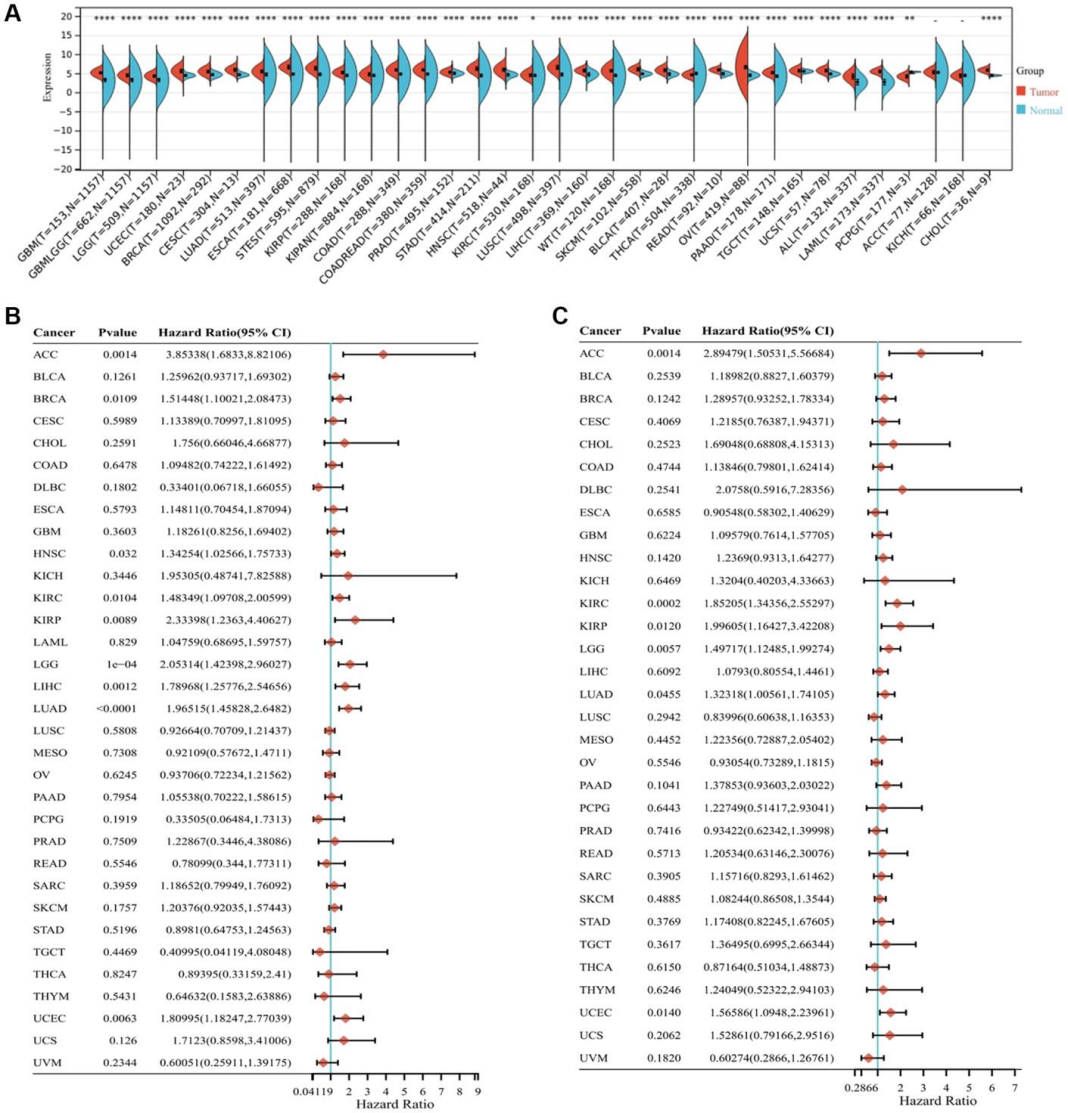
**Expression levels and prognostic analysis of ALG3 in pan-cancer.** (**A**) ALG3 expression difference in across tumors integrating data of normal tissues in GTEx database and data of TCGA tumor tissues, ^*^P < 0.05, ^**^P < 0.01, ^***^P < 0.001, ^****^P < 0.0001. (**B**) Forest plot of the relationship between ALG3 expression and overall survival time. (**C**) Forest plot of the relationship between ALG3 expression and disease-free survival time.

In this study, we combined data on normal tissues derived from the Genotype-Tissue Expression (GTEx) database with that of tumor tissues derived from The Cancer Genome Atlas (TCGA) to examine the differential expression of ALG3 in pan-cancer utilizing the SangerBox database (http://vip.sangerbox.com/). It has been discovered that ALG3 expression levels are usually elevated in nearly all cancers in contrast with that in normal tissues (Figure 1A).

The correlation of ALG3 expression with overall survival (OS) and disease-free survival (DFS) was calculated individually in 33 TCGA cancers utilizing univariate survival analysis. As illustrated in Figure 1B, ALG3 expression markedly impacted the OS of LUAD, LGG, LIHC, ACC, UCEC, KIRP, KIRC, BRCA and HNSC. The correlation of ALG3 expression with PFS was illustrated in Figure 1C, which showed that the DFS duration of patients with LAML, KIRC, ACC, LGG, KIRP, UCEC and LUAD exhibiting elevated ALG3 expression levels was substantially shortened in contrast with that of those exhibiting low ALG3 expression level. In general, the findings showed that ALG3 could be a candidate for use as a biomarker in multiple malignancies.

### Association between ALG3 expression and immune-related cells in pan-cancer

Tumor microenvironment (TME) has been reported to be closely related to the clinical prognosis of cancer patients. Diverse immune cell populations, including innate and adaptive immune cells, such as myeloid cells and lymphocytes within the TME, comprise most of the TME. Thus, we focused on the association between the expression of ALG3 and the particular kinds of immune cells. As a consequence, we examined the link between the expression of the ALG3 gene and the infiltration of diverse immune cells, such as adaptive immune cells (CD8+ T cell, CD4+ T cell, B cell) and innate immune cells (neutrophil and macrophage). Tumor Immune Estimation Resource (TIMER) algorithms were utilized to ascertain the enrichment status of immune cells. Supplementary Figure 1 presented that ALG3 was notably correlated with immune infiltrating cells in LUSC, SKCM, STES, STAD and BRCA.

### Analysis of the correlation between ALG3 and TMB, MSI, and immune checkpoint genes in pan-cancer

TME, MSI and immune checkpoint genes were important indicators for predicting the immune microenvironment. We examined the link between ALG3 and TMB, MSI, and the immune checkpoint genes to determine if the expression of ALG3 is linked to the modulation of the immune conditions. Home-for-researchers online software was used to examine the connection between ALG3 and TMB, MSI.

**Figure 2:**
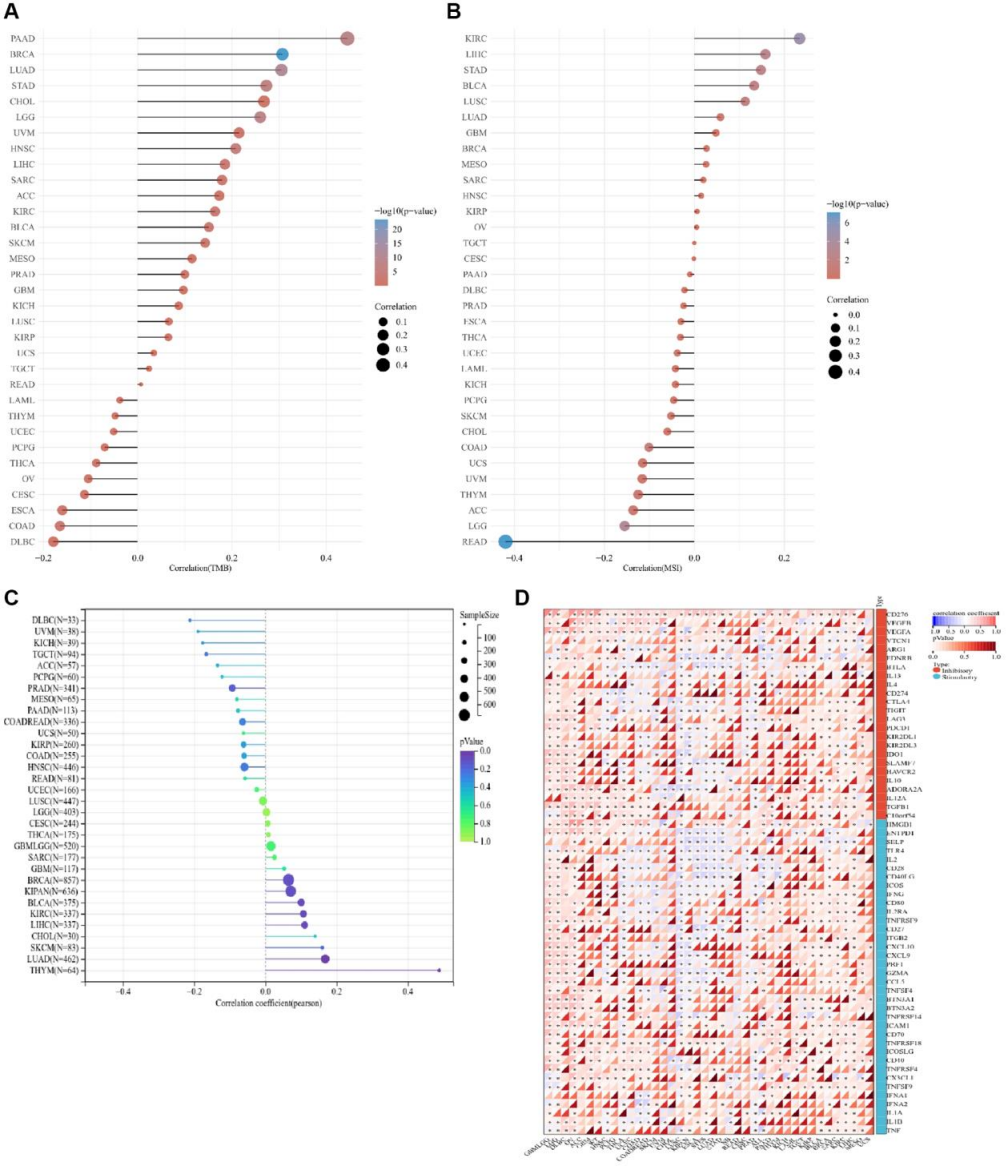
**Correlation analysis between ALG3 and TMB, MSI and immune checkpoint genes in pan-cancer.** (**A**) The correlation between ALG3 expression and TMB in pan-cancer. (**B**) The correlation between ALG3 expression and MSI in pan-cancer. (**C**) The association between ALG3 expression level and neoantigen in pan-cancer. (**D**) The correlation between ALG3 expression and immune checkpoint genes in pan-cancer.

As depicted in Figure 2A and Supplementary Table 1, ALG3 expression was considerably linked to TMB in 14 out of 33 distinct kinds of cancers (BRCA, LUAD, LGG, PAAD, STAD, HNSC, LIHC, COAD, SKCM, KIRC, BLCA, SARC, PRAD and ESCA). In COAD and ESCA, ALG3 expression was inversely linked to TMB but was linked to TMB in other 12 forms of cancers in a positive manner.

As displayed in Figure 2B and Supplementary Table 2, ALG3 expression was considerably related to MSI in READ, KIRC, LGG, LIHC, STAD, BLCA, LUSC and COAD. While ALG3 exhibited a positive link to MSI in READ, LGG and COAD, it had an inverse link to MSI in KIRC, LIHC, STAD, BLCA and LUSC.

Tumor neoantigens, a new approach to tumor immunotherapy, include antigens produced by tumor viruses integrated into the genome and antigens produced by mutant proteins, which are abundantly expressed only in tumor cells and have strong immunogenicity and tumor heterogeneity. A growing number of studies have highlighted the relationship between neoantigens and T cells’ recognition of cancer cells. We also analyzed the association between ALG3 expression level and tumor neoantigen. The result showed that the expression of ALG3 was shown to be positively linked to tumor neoantigen in LUAD and THYM (Figure 2C).

Figure 2D presents the findings from our investigation into whether or not there is a link between the expression of 47 different immune checkpoint genes and ALG3 in 33 different forms of cancer as determined with the help of Sangerbox. Collectively, the expression of ALG3 was shown to be positively linked to the expression of genes associated with immune checkpoints in the majority of cancers, which supported the assumption that ALG3 might be effective in tumor immunotherapy.

### ALG3 predictive value in response to immune checkpoint blockade (ICB)

**Figure 3:**
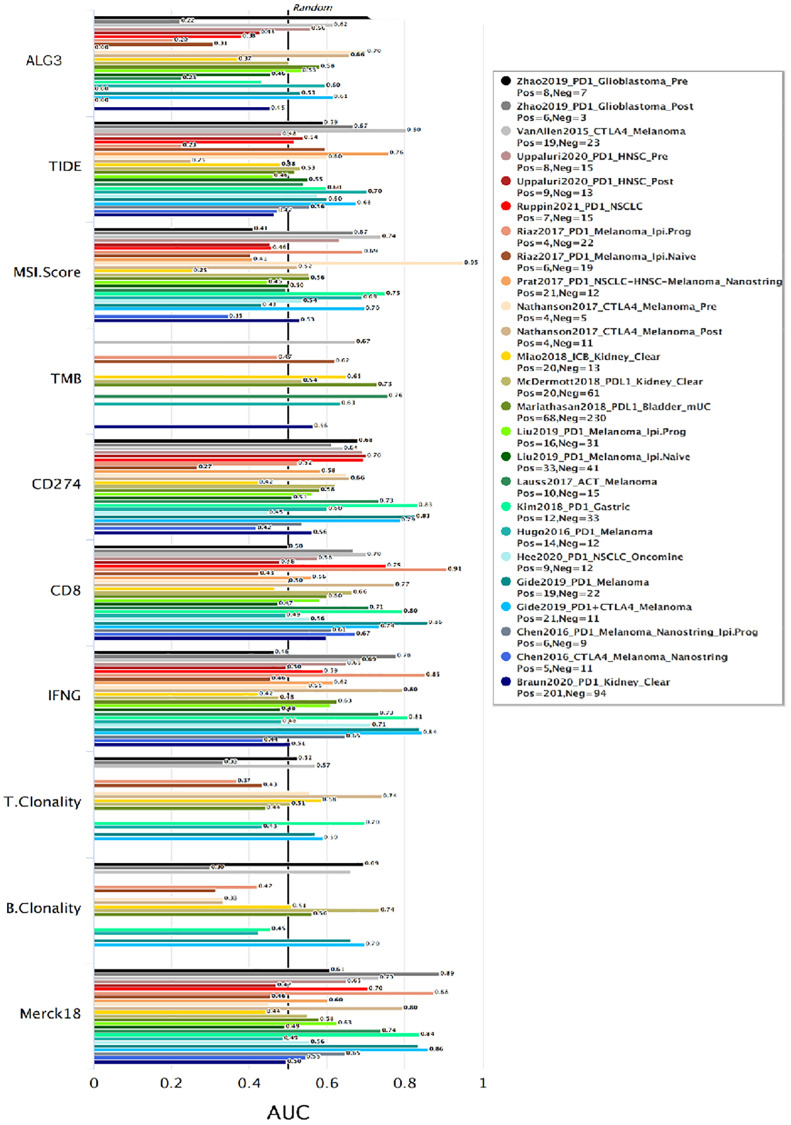
**ALG3 predictive value in response to immune checkpoint blockade (ICB).** Bar plot showing the biomarker relevance of ALG3 compared to standardized cancer immune evasion biomarkers in immune checkpoint blockade (ICB) sub-cohorts. The area under the receiver operating characteristic curve (AUC) was applied to evaluate the predictive performances of the test biomarkers on the ICB response status.

The above data implicated that ALG3 may be an effective indicator for immune checkpoint blockade. We evaluated the significance of ALG3 as a biological marker by contrasting its levels of expression with that of standardized biomarkers premised on the prediction performance of response outcomes and OS of ICB sub-cohorts. This was done by utilizing the Tumor Immune Dysfunction and Exclusion database (http://tide.dfci.harvard.edu/). Notably, we discovered that ALG3 independently exhibited an area under the receiver operating characteristic curve (AUC) of >0.5 in 11 of the 21 ICB cohorts. ALG3 exhibited a higher predictive value than TMB, T. Clonality, and B. Clonality. However, ALG3 was comparable to the MSI score, CD274, CD8, IFNG, TIDE and Merck18 in predication (Figure 3).

### ALG3 genes are linked to tumor stemness and chemo-therapy susceptibility of cancer cells

**Figure 4:**
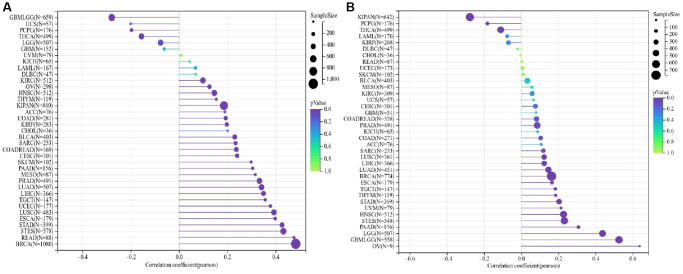
**ALG3 genes are associated with tumor stemness and cancer cell sensitivity to chemotherapy.** (**A**, **B**) Correlation matrix between ALG3 gene expression and cancer stemness scores RNAss and DNAss, respectively.

During the progression of cancer, tumor cells may lose their differentiated phenotype steadily and gain characteristics similar to those of progenitor and stem cells. Both RNA stemness score, which is premised on mRNA expression (RNAss), and DNA stemness score (DNAss), which is dependent on the patterns of DNA methylation, may be utilized to quantify the stemness of a tumor. DNAss is the dryness index derived based on methylation data while RNAss is the stemness indices calculated based on expression data. When the stemness index is closer to 1, the degree of cell differentiation tends to be lower, whereas the stem cells’ characteristics become stronger. We examined whether or not there was a link between the ALG3 genes and the stemness of the tumor, as estimated by DNAss and RNAss. ALG3 was shown to have varying degrees of relationship with RNAs and DNAs, depending on the kind of cancer (Figure 4A, 4B).

Because cancer stem cell often contributes to drug-resistance, we thus explored the association between ALG3 gene expression and drug resistance. With the use of RNAactDrug database (http://bio-bigdata.hrbmu.edu.cn/RNAactDrug), we found that elevated ALG3 expression level was linked to resistance to 1,6-bis(4-(4-aminophenoxy)phenyl)diamantine and sb-590885-aad. However, elevated ALG3 expression level predicated sensitivity to CI-1040, Olaparib, Trametinib and VX-11e (Supplementary Table 3).

### Association with ALG3 and clinical-pathological parameters, and prognostic significance in breast cancer

**Figure 5:**
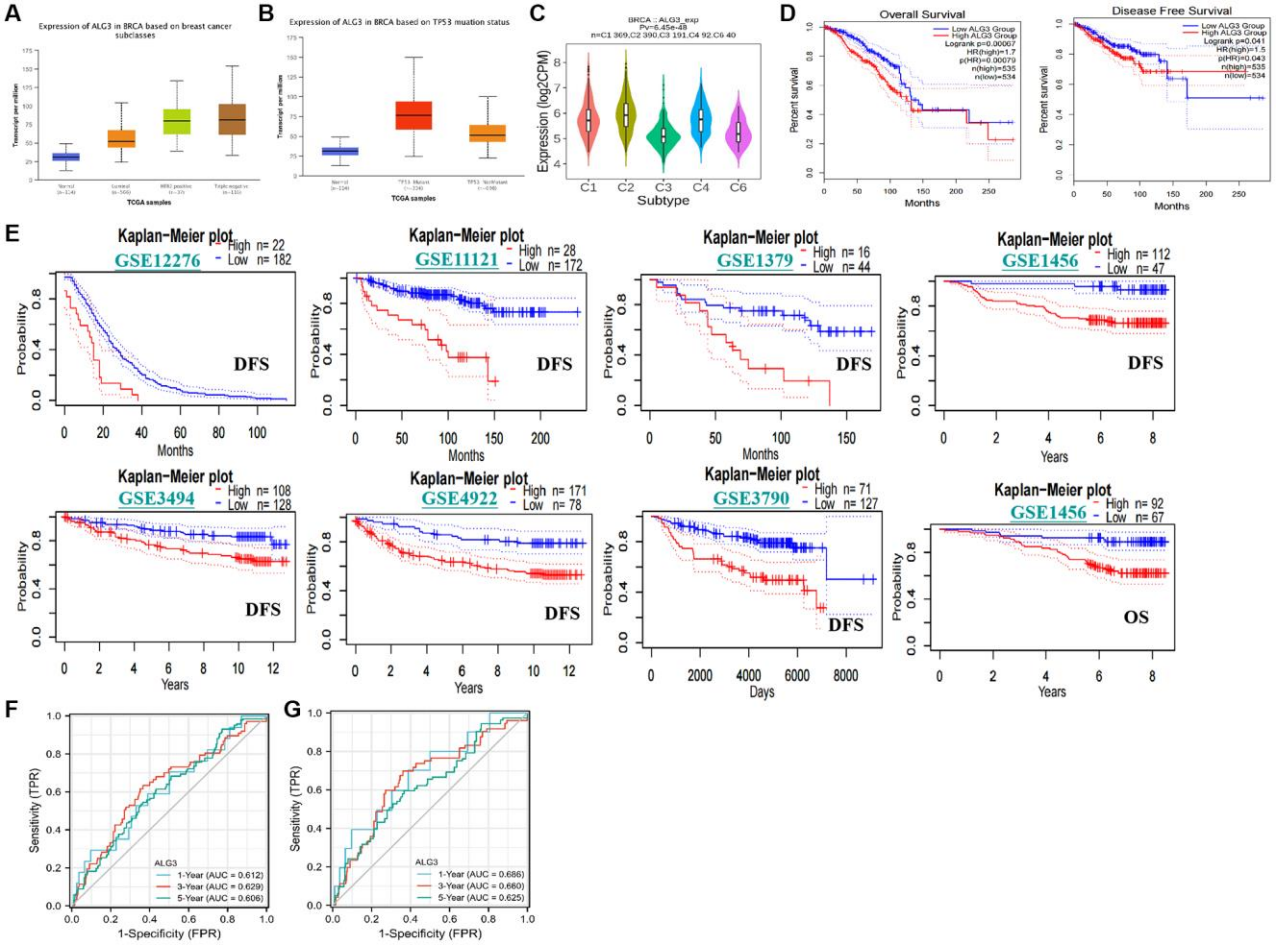
**Association with ALG3 and clinico-pathological characters, prognostic value in breast cancer.** (**A**) Expression levels of ALG3 were indicated in normal breast tissues and different sub-type of breast cancer tissues. (**B**) Association between expression level of ALG3 and TP53 mutant status was demonstrated. (**C**) The association between expression immune subtypes and expression level of ALG3. C1 (wound healing); C2 (IFN-gamma dominant); C3 (inflammatory); C4 (lymphocyte depleted); C5 (immunologically quiet); C6 (TGF-β dominant). (**D**) TCGA database revealed that high expression level of ALG3 in breast cancer patients predicated poorer OS and DFS rates. (**E**) The GEO databases revealed that high expression level of ALG3 in breast cancer patients predicated poorer OS and DFS rates. (**F**, **G**) ROC analysis for prediction of ALG3 for OS and DFS, respectively.

Subsequently, we evaluated whether ALG3 expression level was correlated with clinical-pathological parameters, and prognostic significance. The elevated expression level of ALG3 was found in breast cancer, especially in HER-2 positive and triple negative breast cancer, as revealed by the UALCA database (Figure 5A). An elevated level of ALG3 expression was considerably linked to TP53 mutant status (Figure 5B).

The 6 distinct immune subtypes, namely, C1 (wound healing), C2 (IFN-c dominant), C3 (inflammatory), C4 (lymphocyte depleted), C5 (immunologically quiet), and C6 (TGF-β dominant) have been well recognized. Individuals whose immune subtypes were C3 or C5 exhibited a favorable prognosis, but those whose immune subtypes were C4 or C6 exhibited a much lower chance of survival. We evaluated the link between the level of ALG3 expression and the immunological subtype based on the TISIDB database (http://cis.hku.hk/TISIDB/). Breast cancer patients exhibiting subtypes C1, C2, C4, and C6 were shown to have elevated expression levels of ALG3, suggesting that ALG3 may have a role in promoting the growth of tumors (Figure 5C).

We investigated the potential role of ALG3 as a predictive factor in breast cancer patients based on data derived from the TCGA database. The median value of ALG3 was used as the threshold to classify patients with breast cancer into high- and low-expression groups. It was found that elevated expression level of ALG3 was linked to poorer OS rate and DFS rate (Figure 5D). In parallel, we further confirmed that ALG3 expression was inversely linked to breast cancer patients OS and DFS in the GEO database, by utilizing the PrognoScan database (http://dna00.bio.kyutech.ac.jp/PrognoScan/index.html) (Figure 5E).

Subsequently, the time receiver operating characteristic (ROC) analysis was carried out to compare the prediction power and risk score of ALG3 for OS and DFS in breast cancer. In the OS analysis, ALG3 expression level could anticipate the breast cancer patients’ prognoses over 1, 3, and 5 years, and its AUC values were 0.612, 0.629, and 0.606, correspondingly (Figure 5F). In the PFS analysis, AUC values were 0.686, 0.660 and 0.625, correspondingly (Figure 5G).

Finally, the multivariate and univariate Cox regression analyses were executed to establish the prognostic relevance of ALG3 in breast cancer. The findings recorded from the univariate analysis highlighted that T, N, M stage and ALG3 expression levels were substantially linked to OS rate in breast cancer patients (Table 1). On the contrary, the multivariate Cox regression analysis illustrated that N, M stage and ALG3 expression levels were substantially linked to OS rate in LUAD patients (Table 1).

**Table 1:** Summary of univariate and multivariate Cox regression analysis of overall survival duration.

**Characteristics**	**Total (*N*)**	**Univariate analysis**		**Multivariate analysis**	
		**Hazard ratio (95% CI)**	***P*-value**	**Hazard ratio (95% CI)**	***P*-value**
T stage	1079				
T1 and T2	905				
T3 and T4	174	1.608 (1.110–2.329)	**0.012**	1.381 (0.915–2.086)	0.125
N stage	1063				
N0	514				
N1, N2 and N3	549	2.239 (1.567–3.199)	**<0.001**	1.817 (1.235–2.671)	**0.002**
M stage	922				
M0	902				
M1	20	4.254 (2.468–7.334)	**<0.001**	2.454 (1.337–4.504)	**0.004**
ALG3	1082				
Low	541				
High	541	1.565 (1.134–2.161)	**0.006**	1.669 (1.170–2.382)	**0.005**

### scRNA analysis of ALG3 expression on distinct cells of breast cancer

**Figure 6:**
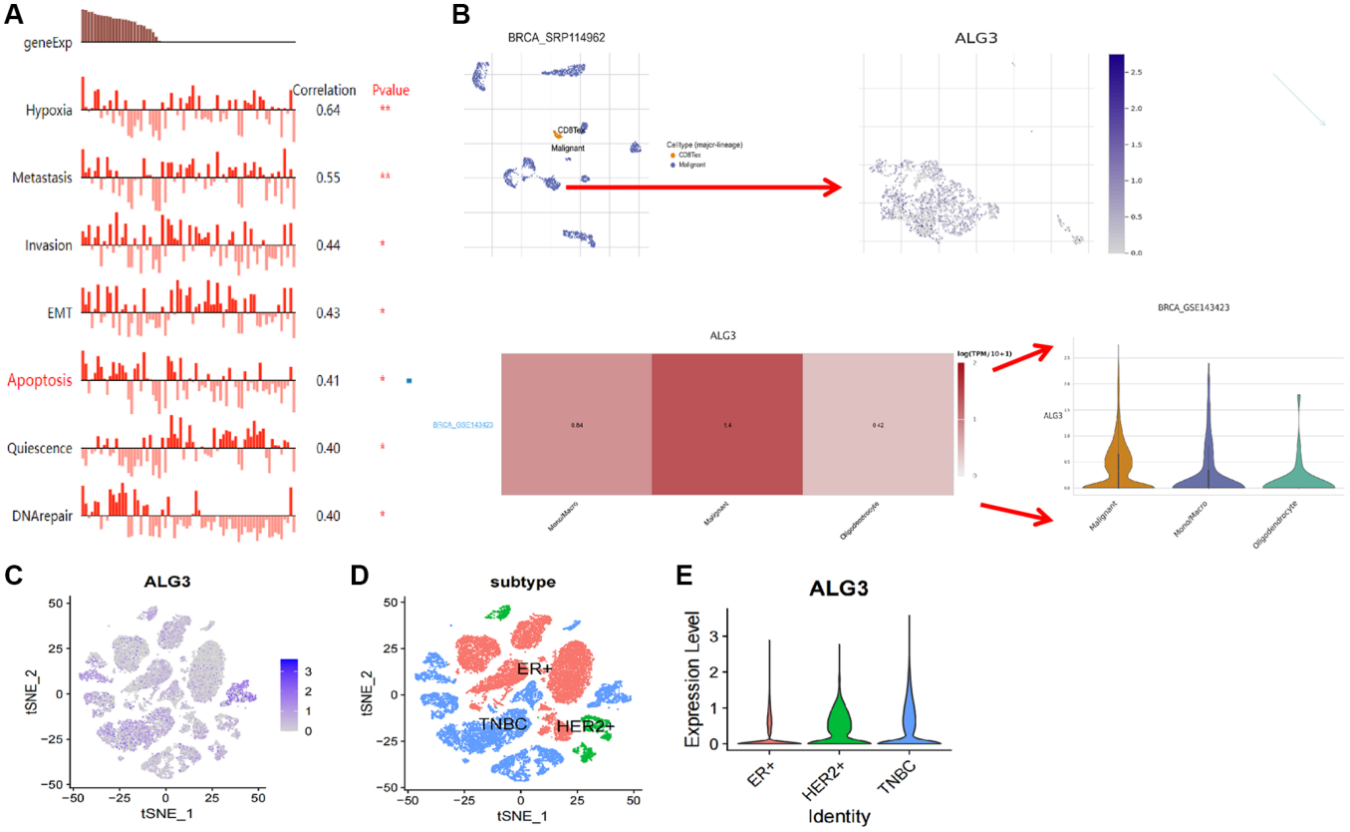
**ScRNA analysis of ALG3 expression on different cells of breast cancer.** (**A**) Functional pathways involved in ALG3, revealed by CANCERSEA website. (**B**) The analysis of the expression of PUDP on the cell in the tumor microenvironment. (**C**) Log-normalized expression of markers for ALG3 in breast cancer. (**D**, **E**) ALG3 expression level in different clinical subtype was demonstrated.

The CANCERSEA website (http://biocc.hrbmu.edu.cn/CancerSEA/) was employed to validate the expression of ALG3 in single-cell and its link to tumor functional state in breast cancer. Data from Jordan NV et al. revealed that ALG3 may participate in hypoxia, metastasis, invasion, EMT, apoptosis, quiescence, and DNA repair (Figure 6A).

We further used the findings of breast scRNA sequencing from the GSE143423 dataset to examine the ALG3 expression in diverse cells of breast cancer by using the online software Tumor Immune Single-cell Hub (TISCH) (http://tisch.comp-genomics.org/home/). We discovered that ALG3 was expressed in monocytes or macrophages, oligodendrocytes, and particularly in tumor cells exhibiting considerably elevated expression levels (Figure 6B). The findings implicated that ALG3 expression was related to the tumor microenvironment.

Furthermore, we used GSE152048 to examine the ALG3 expression in diverse cells of breast cancer. Figure 6C showed the distribution of ALG3 in breast cancer clusters. Interestingly, we found that the level of ALG3 was often elevated in HER-2+ and TNBC, which had a higher degree of malignancy than ER+ breast cancer (Figure 6D, 6E).

### Spatially mapping the association of ALG3 expression level and significant pathways in breast cancer

**Figure 7:**
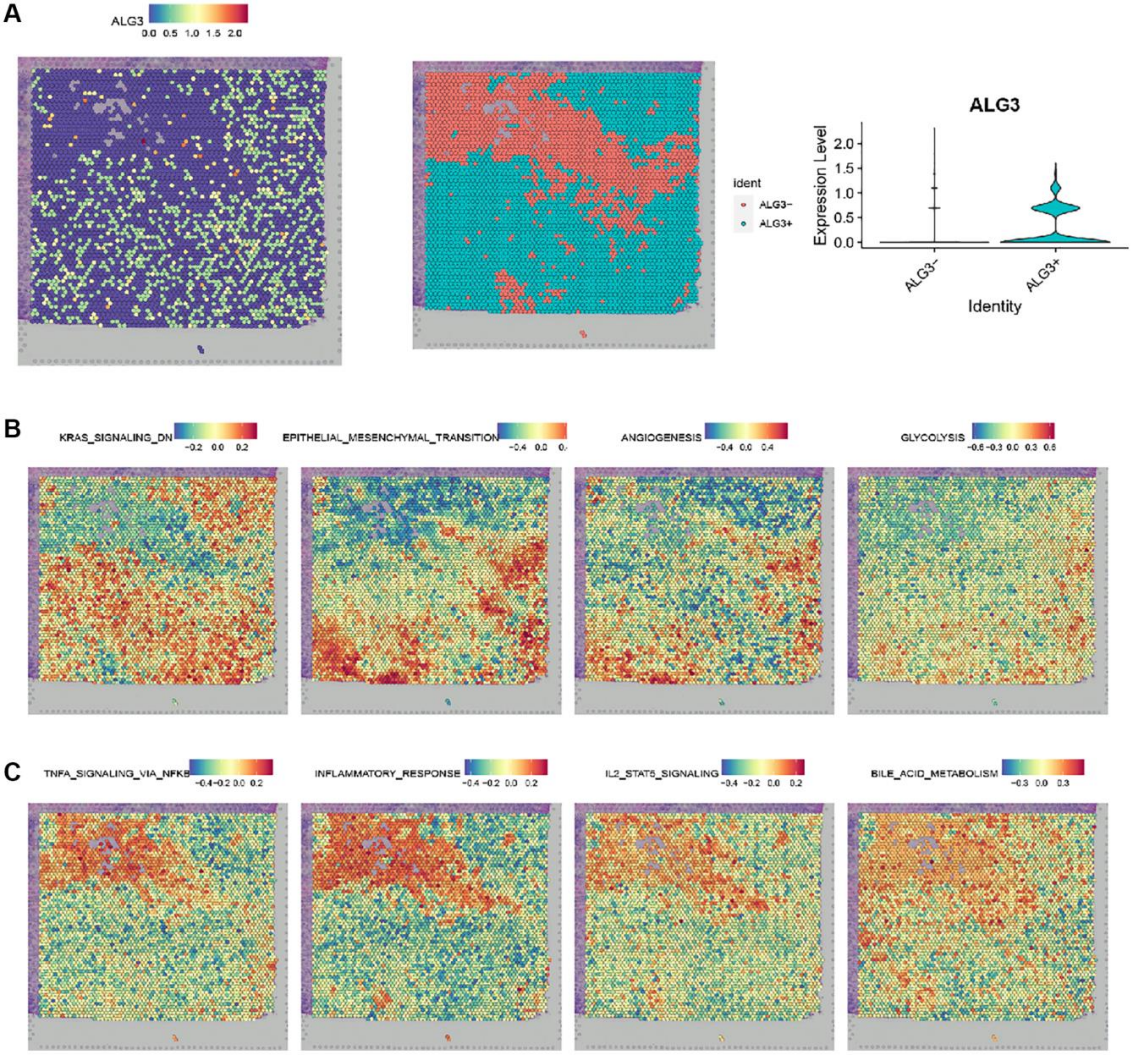
**Spatially mapping the association of ALG3 expression level and significant pathways in breast cancer.** (**A**) Spatial plots showing the expression level of ALG3. (**B**) Spatial plots showing spatial spots colored by subclusters. (**C**) Spatial plots showing GSVA scores of pathways.

To gain insights into the association of ALG3 expression level and significant pathways, we performed spatially resolved transcriptomics on breast cancer samples. Figure 7A showed the expression level of ALG3 in breast cancer samples. The subclusters was identified as ALG3+ and ALG3-, as shown in Figure 7B. The analysis revealed that ALG3 was positively associated with the GSVA scores of pathways that were involved in breast cancer progression, such as KRAS signaling, EMT, TNF α signaling pathways. Interestingly, ALG3 was positively associated with the GSVA scores of bile acid and glycolysis metabolism (Figure 7C).

### ALG3 expression is linked to treatment responsiveness in breast cancer

**Figure 8:**
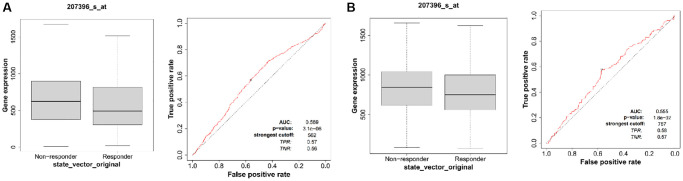
**ALG3 expression is associated with therapeutic responses in breast cancer.** (**A**) The receiver operating characteristic (ROC) curve plot of the association between ALG3 expression and responses to chemotherapy in breast cancer who had pathology complete response. (**B**) The ROC curve plot of the association between ALG3 expression and responses to chemotherapy in breast cancer who had relapse-free survival at five years.

We assessed the impact of ALG3 expression on susceptibility to chemotherapy in clinical cancer cohorts premised on the ROC plotter database (https://www.rocplot.org/). In breast cancer patients who had pathology complete response, elevated expression levels of ALG3 were resistant to the chemotherapeutics (Figure 8A). Similar results were achieved in breast cancer patients who had relapse-free survival at five years (Figure 8B). However, we did not observe significant correlation between ALG3 expression levels and endocrine therapy or anti-Her-2 therapy in breast cancer patients (data not shown).

### Inhibition of ALG3 decreased breast cell growth

We further performed a functional assay to explore ALG3’s function in breast cancer cells.

**Figure 9:**
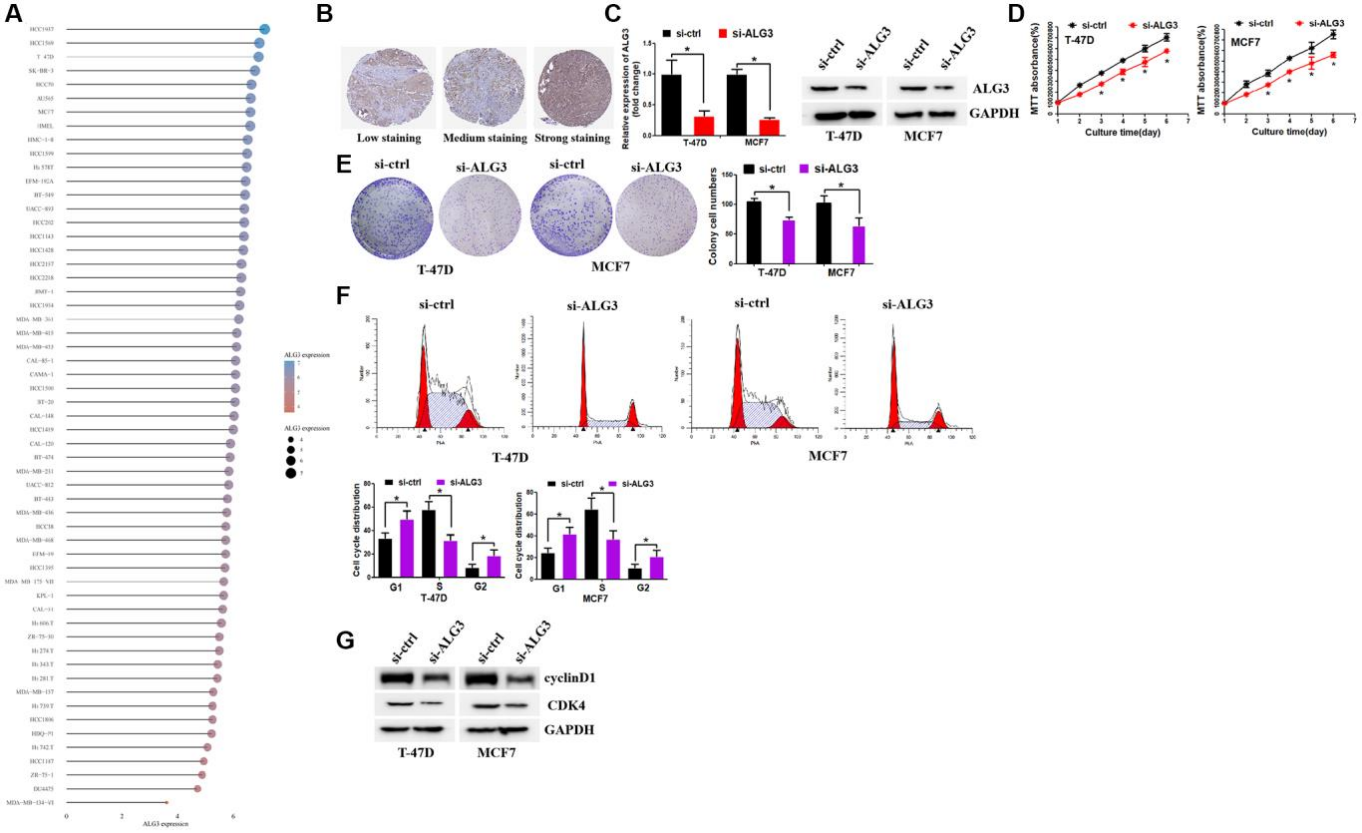
**Inhibition of ALG3 decreased breast cell growth.** (**A**) CCLE database demonstrated ALG3 expression levels in breast cancer cell lines. (**B**) The expression of ALG3 in breast cancer tissues on the level of protein. (**C**) RT-PCR and Western blot assays were used to examine si-RNA efficiency in breast cancer cells (left panel: RT-PCR; right panel: Western blot). (**D**) MTT assay was used to examine ALG3 down-regulation effect on breast cancer cells. (**E**) ALG3 down-regulation inhibited breast cancer cell colony formation ability. (**F**) Suppression of ALG3 contributed to the arrest of the cell cycle in the G1 and G2 phase. (**G**) Western blot assay was used to examine the expression levels of cyclinD1 and CDK4.

With the use of the CCLE database, we revealed the elevation of ALG3 expression in most kinds of breast cancer cell lines (Figure 9A). In addition, we revealed that ALG3 expression was elevated in breast cancer samples by utilizing Human Protein Atlas (HPA) database (Figure 9B). Inhibition of ALG3 expression was achieved by using si-RNA that targets ALG3 (Figure 9C). ALG3 inhibition decreased breast cancer cell growth ability, as revealed by MTT and colony formation assays (Figure 9D, 9E). We next explored whether ALG3 contributed to cell growth by affecting cell cycle distribution. It was found that suppression of ALG3 contributed to the arrest of the cell cycle in the G1 and G2 phase (Figure 9F). Western blot assay revealed that inhibition of ALG3 decreased the expression levels of cyclinD1 and CDK4 (Figure 9G). CyclinD1 and CDK4 were key kinase that regulated cell cycle distribution and affected cell proliferation. These data implicated that ALG3 may promote breast cancer proliferation via regulating cell cycle kinase-cyclinD1 and CDK4.

## DISCUSSION

Owing to rapid advances in medical technology and the persistent research efforts of oncologists, the diagnosis, monitoring, and treatment of oncological illnesses are becoming more methodical, all-encompassing, and individualized [17, 18]. Not with standing this, there is no limit to the number of tailored and successful cancer therapies that might be discovered.

ALG family represents a class of glycosyltransferases [19, 20]. In esophageal squamous cell carcinoma, ALG3 was shown to be a viable treatment target, and its overexpression was revealed to be closely related to lymph node metastasis [5]. An elevated level of ALG3 expression in breast cancer patients is predictive of a dismal prognosis because it stimulates the proliferative and metastatic capacities of cancer cells [5]. Although previous studies have implicated that ALG3 was involved in breast cancer progression, whether ALG3 functioned as an onco-immunological biomarker in breast cancer was still unknown. We focused our attention on determining the significant role that ALG3 plays in the progression of a variety of malignancies by meticulously examining the data that was gathered from the GTEX and CCLE databases from a large number of cases of diverse kinds of cancer. We started by examining the aberrant expression of ALG3 in a variety of malignancies. We employed Cox and KM curve analyses and discovered that various survival markers had varying expression profiles of ALG3. This prompted us to infer that ALG3 has potential prognostic significance in some types of cancers.

The comprehensive treatment of tumors is continuously being refined, and the available management options comprised surgery, chemotherapy, endocrine therapy, and targeted therapy [21]. The field of oncology is now focusing a lot of attention on immunotherapy as a way to aid in the selection of appropriate treatment. Combination immunotherapy is a viable method that should be pursued. Its primary objective is to repair the immunologic deficiencies that are present in the TME [22, 23]. Tumor-infiltrating lymphocytes (TIL) perform a significant part in improving clinical outcomes and eliciting a response to treatment in patients with diverse forms of cancer [24, 25]. In this research, we discovered that ALG3 is capable of efficiently activating all 6 distinct kinds of immune cells (neutrophils, CD8+ T cells, macrophages, CD4+ T cells, dendritic cells, and B cells) in approximately 20 different types of cancer. Additionally, immune biomarkers had also been linked to ALG3 expression in most kinds of cancer.

Both the prognosis and the patient’s response to therapy may be anticipated based on particular gene mutations and MSI [26]. Increased somatic MSI and TMB were shown to be connected to enhanced efficacy of immunotherapy as well as a greater OS for the majority of cancer individuals [27]. The upstream causes of MSI were determined to be mutations in mismatch repair genes as well as an impaired function in those genes. We discovered that ALG3 was linked to TMB in 14 out of 33 distinct kinds of cancers (BRCA, LUAD, LGG, PAAD, STAD, HNSC, LIHC, COAD, SKCM, KIRC, BLCA, SARC, PRAD and ESCA). In addition, we also found that ALG3 was considerably related to MSI in READ, KIRC, LGG, LIHC, STAD, BLCA, LUSC and COAD. All of these findings indicate that ALG3 could promote tumor growth by increasing MSI and TMB via the mechanism of modulating genes involved in mismatch repair.

The so-called cancer stem cells (CSCs) are a subset of cancerous cells that are extremely tumorigenic with strong resistance to a variety of therapeutic approaches [28]. It is now well-established that CSCs perform an integral function in the growth of tumors and in the development of resistance to treatment [29]. The cancer stem cell-like characteristics evaluated by mRNA (RNAss) and DNA methylation (DNAss) exhibited a substantial inverse link to ALG3, as shown by the analysis results. Based on these findings, it is conceivable that ALG3 is implicated in the production of tumor-initiating cells and has a connection to the development of drug resistance in tumors. The link between ALG3 and a patient’s treatment susceptibility to anti-cancer medications was investigated in this research for the first time. Elevated ALG3 expression level was linked to resistance to 1,6-bis(4-(4-aminophenoxy)phenyl)diamantine and sb-590885-aad. However, elevated ALG3 expression level was associated with sensitivity to CI-1040, Olaparib, Trametinib and VX-11e.

The biological function and the fundamental processes of ALG3 in breast cancer were investigated. A single-cell transcriptome sequencing investigation illustrated that ALG3 expression was substantially related to hypoxia, metastasis, invasion, EMT, apoptosis, quiescence, and DNA repair in breast cancer. The inhibition of ALG3 activity resulted in a decrease in the growth rate of breast cancer cells.

Throughout the process of breast cancer progression, the cancerous cells interface with other cells, including endothelial cells and macrophages, to establish the milieu that supports the growth and metastasis of cancer cells. Tumor-associated macrophages, also referred to as TAMs, are a type of immune effector cells recruited to carcinoma tissues so that they may secrete a variety of chemokines, growth factors, cytokines, and inflammatory mediators, all of which perform a significant part in the progression of cancer [30]. Altogether, we have investigated the relationships that exist between the expression of ALG3 and a wide range of cells and molecules that are associated with tumors. After that, we focused on breast cancer in an effort to gain a greater comprehension of ALG3-elicited intercellular interactions as well as the activation of signal transduction pathways. According to the findings of this research, ALG3 is a major contributor to the onset and progression of a wide variety of cancer types, and the targeted suppression of ALG3 could be a useful method for treating cancer.

In summary, we made comprehensive analysis of ALG3 in pan-cancer and validation of ALG3 as an onco-immunological biomarker in breast cancer. These points were the innovation and importance of our research.

## MATERIALS AND METHODS

### Cell culture

Breast cancer cell lines (T-47D and MCF7) were purchased from the Chinese Academy of Science (Shanghai, China). Cells were maintained in DMEM supplemented with 10% fetal bovine serum (FBS) in a 37°C, 5% CO_2_ incubator.

### Analysis of ALG3 expression in pan-cancer

To investigate the expression of the ALG3 gene, we gathered information from tumor cell lines collected from the Cancer Cell Line Encyclopedia (CCLE) database and information about normal tissues from the GTEx database. In addition, we employed the data derived from TCGA and the data from the GTEx database that corresponded to normal tissue to examine the variation in expression levels that exist between tumor and normal samples.

### Survival analysis

Utilizing clinical information contained in the TCGA database, we selected two markers (OS and PFS) to characterize the survival duration and prognosis of patients with a variety of malignancies. Estimating the association between ALG3 levels and patients’ prognoses utilizing forest plots was demonstrated to be effective. Eventually, survival analysis was implemented utilizing Kaplan–Meier curves, which were applied to the data of certain malignancies that had statistical significance.

### Co-expression analysis

An examination of the association between ALG3 and the expression of immune checkpoints was done utilizing Pearson’s method. After creating the correlation heatmaps, each illustrated the state of the ALG3-associated co-expression. By employing the online analysis platform HOME for Research (http://www.home-for-researchers.com/), we explored the link between MSI, TMB, and ALG3 expression across multiple-typed cancers.

### Cell infiltration analysis

We employed the Estimation of stromal and Immune cells in Malignant Tumor tissues using the Expression data (ESTIMATE) algorithm to investigate the possible correlation between the immune and stromal cells and ALG3 expression in the TME. Calculations of *p*-values and partial correlation values were carried out with the help of Spearman’s rank correlation test. Scatter plots were used to illustrate the findings. In addition, we selected ALG3 highly-relevant malignancies to analyze the density distribution, which was subsequently depicted using distribution maps. We utilized a TIMER (https://cistrome.shinyapps.io/timer) database to evaluate the specific infiltration status of various immune cells, such as CD8+ T cells, neutrophils, CD4+ T cells, B cells, macrophages, and dendritic cells, and their connection toALG3. Scatter plots were utilized to display the data.

### scRNA-seq analysis

Single-cell RNA sequencing, abbreviated as scRNA-seq (GSE143423 and GSE152048), were utilized to find out the expression and distribution of ALG3 expression in breast cancer patients. After obtaining the data of scRNA-seq, analysis was conducted with the help of TISCH.

### Spatial transcriptomics and visium spatial transcriptomics data

All spatially resolved transcriptomics data from this study are available from the Zenodo data repository (https://doi.org/10.5281/zenodo.4739739). Spatially resolved transcriptomics data from Andersson et al. can be downloaded from the Zenodo data repository (https://doi.org/10.5281/zenodo.3957257). The Space Ranger software v.1.0.0 (10x Genomics) was used to demultiplexed reads and mapped them to the reference genome GRCh38. Count matrices were loaded into Seurat v.3.2.0 and STutility v.0.1.0 for all subsequent data filtering, normalization, filtering, dimensional reduction and visualization. Data normalization was performed on independent tissue sections using the variance-stabilizing transformation method implemented in the SCTransform function in Seurat.

### Cell cycle analysis

The treated cells were collected and fixed with chilled 75% ethanol at −20°C overnight. After ethanol was discarded, cells were washed twice with PBS and resuspended with DNA staining solution at room temperature for 30 min. Cell cycle analysis was performed on the flow cytometry.

### Real-time PCR

After isolating the total RNA from cells and tissues, a FastKing RT Kit (TianGen, Beijing, China) was utilized to perform reverse transcription of these RNAs into cDNA. Afterward, real-time PCR was implemented with the help of SYBR Premix ExTaqTMa (RR420A, TaKaRa, Japan) following the guidelines provided by the manufacturer. Primers for ALG3 were: forward (5′-CCGAGGTAGAAGGCGTCATC-3′); reverse (5′-GGTACACAAGTGGTCCGGT-3′). Primers for GAPDH were: forward (5′-GGAGCGAGATCCCTCCAAAAT-3′); reverse (5′-GGCTGTTGTCATACTTCTCATGG-3′).

### The levels of ALG3 protein expression in breast cancer patients

The Human Protein Atlas (HPA, https://www.proteinatlas.org/), which is an initiative based in Sweden, provides all of the proteins found in humans. We retrieved the immunohistochemical (IHC) images of ALG3 expression that are publicly accessible for all research communities from the HPA database.

### Western blot analysis

The total cellular proteins from cells were extracted by using RIPA lysis buffer with phenylmethanesulfonyl fluoride (PMSF). The equal amounts of protein were determined by BCA protein assay kit, followed by separated with 10% SDS-PAGE gels. The proteins were then transferred to PVDF membranes and blocked with 5% nonfat milk. Subsequently, the PVDF membranes were subjected to corresponding primary anti-bodies and secondary anti-bodies. Investigations utilizing Western blotting were conducted with an electrophoresis and blotting system (Bio-Rad, USA) as per the recommendations provided by the manufacturer.

### MTT assay

For MTT assay, cells were seeded into a 96-well plate per well with three duplications, followed by incubation for 2 h at 37°C. Absorbance was detected at 450 nm daily for 4 consecutive days.

### Statistical analysis

The K-M survival analysis was done utilizing the log-rank test. The R packages “survival ROC” and “pROC” were utilized to chart the ROC curve and the AUC. We employed the Spearman statistical approach to ascertain the link between co-expressed genes, immune cells, and the level of ALG3 expression. R software (version: 4.1.0) was utilized to conduct all analyses of statistical data.

### Data sharing statement

The datasets used and/or analyzed during the current study are available from the corresponding author on reasonable request.

## Supplementary Materials

Supplementary Figure 1

Supplementary Tables
