# Supplementary material for: Comprehensive analysis of ALG3 in pan-cancer and validation of ALG3 as an onco-immunological biomarker in breast cancer
Source: Aging (Albany NY). 2024 Feb 7;16(3):2320–39. doi: 10.18632/aging.205483 (PMC10911369; doi:10.18632/aging.205483)
Supplement: Supplementary Figure 1 [file aging-16-205483-s001.pdf]

SUPPLEMENTARY FIGURE

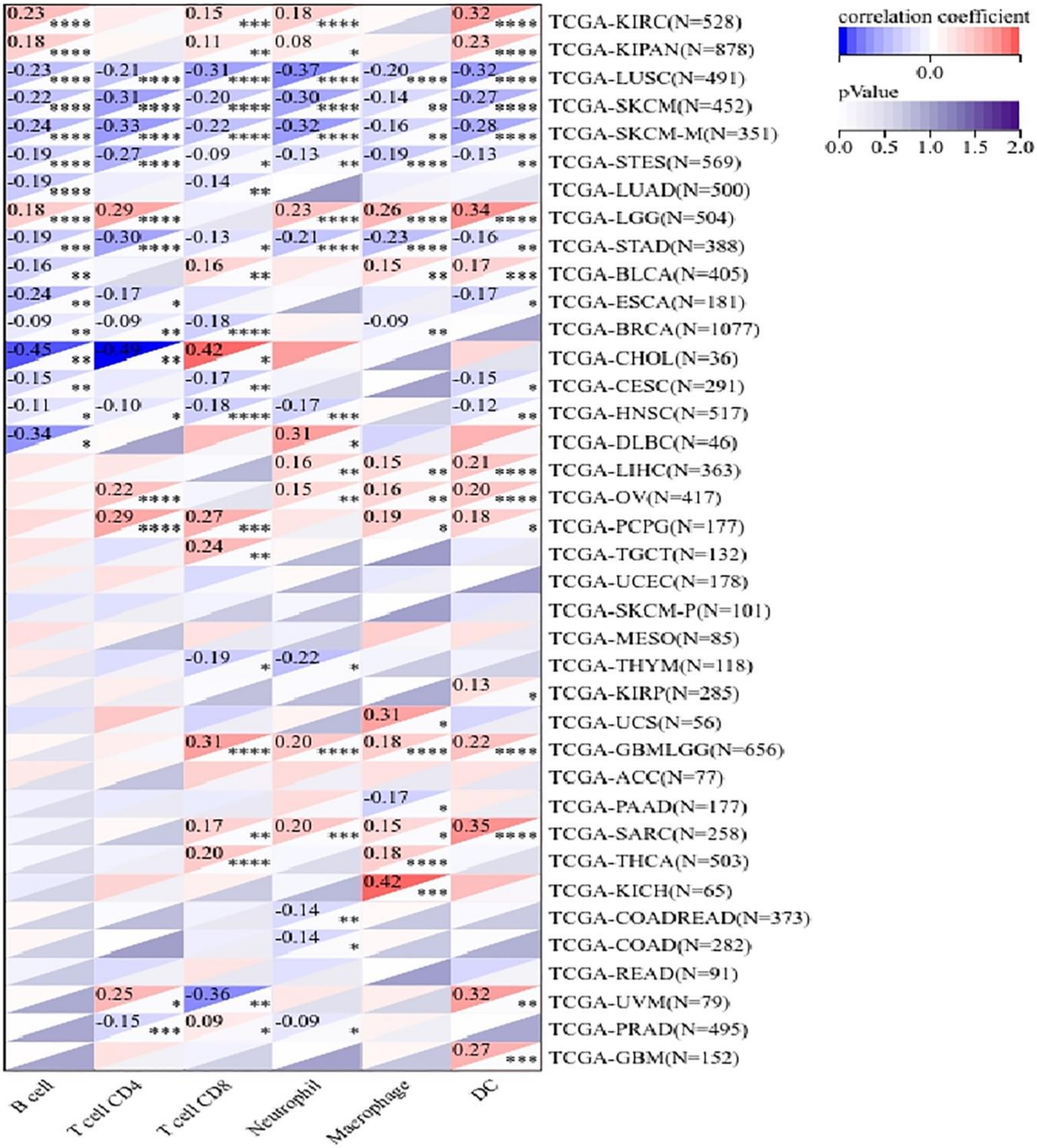

Supplementary Figure 1. Estimation of ALG3-related tumor purity (demonstrated by ESTIMATE Scores) in the tumor microenvironments across cancers in TGCA via ESTIMATE algorithm was investigated. Spearman's *p*-values and partial correlation values are displayed.
