# Supplementary material for: Comprehensive analysis of ALG3 in pan-cancer and validation of ALG3 as an onco-immunological biomarker in breast cancer
Source: Aging (Albany NY). 2024 Feb 7;16(3):2320–39. doi: 10.18632/aging.205483 (PMC10911369; doi:10.18632/aging.205483)
Supplement: Supplementary Tables [file aging-16-205483-s002.pdf]

SUPPLEMENTARY TABLES

Supplementary Table 1. The correlation between ALG3 expression and TMB in pan-cancer.

Gene1	cor	*p*-value
BRCA	0.307	3.59E-24
LUAD	0.305	3.18E-12
LGG	0.26	3.24E-09
PAAD	0.445	4.72E-09
STAD	0.273	1.15E-07
HNSC	0.208	3.76E-06
LIHC	0.185	0.000987
COAD	−0.165	0.00194
SKCM	0.143	0.001964
KIRC	0.164	0.001965
BLCA	0.151	0.002388
SARC	0.179	0.006826
PRAD	0.1	0.028153
ESCA	−0.16	0.048336
THCA	−0.088	0.052552
UVM	0.215	0.055839
OV	−0.105	0.096102
CHOL	0.268	0.119015
ACC	0.173	0.128777
LUSC	0.066	0.154993
CESC	−0.113	0.17105
GBM	0.097	0.236941
UCEC	−0.051	0.250991
KIRP	0.065	0.284375
DLBC	−0.179	0.288619
MESO	0.115	0.306662
PCPG	−0.07	0.357375
KICH	0.087	0.4956
THYM	−0.048	0.606539
LAML	−0.038	0.698572
TGCT	0.024	0.791313
UCS	0.034	0.80003
READ	0.007	0.941038

Supplementary Table 2. The correlation between ALG3 expression and MSI in pan-cancer.

Gene1	cor	*p*-value
READ	−0.42	8.16E-08
KIRC	0.234	1.46E-05
LGG	−0.155	0.000444
LIHC	0.158	0.00234
STAD	0.148	0.00406
BLCA	0.133	0.007216
LUSC	0.113	0.011966
COAD	−0.101	0.036907
THYM	−0.125	0.173987
LUAD	0.058	0.186825
ACC	−0.136	0.232332
SKCM	−0.052	0.257731
UVM	−0.116	0.30445
BRCA	0.027	0.373871
UCEC	−0.038	0.378115
UCS	−0.115	0.394086
THCA	−0.031	0.495778
PCPG	−0.046	0.536034
GBM	0.048	0.555702
PRAD	−0.024	0.587681
LAML	−0.042	0.658811
ESCA	−0.03	0.706082
CHOL	−0.06	0.732751
KICH	−0.042	0.741147
HNSC	0.015	0.745897
SARC	0.02	0.747498
MESO	0.026	0.813552
DLBC	−0.022	0.880927
PAAD	−0.01	0.891779
KIRP	0.006	0.914698
OV	0.005	0.927708
CESC	−0.001	0.991612
TGCT	0	0.996691

Supplementary Table 3. Association between ALG3 expression level and drug sensitivity.

Compound	RNAtype	RNAmolecule	Omics	Source	T. stat	T. fdr	Chi. stat	Chi. fdr	Pearson. stat	Pearson. fdr	Spearman. stat	Spearman. fdr
PD-0325901	mRNA	ALG3	Expression	CCLE	–	–	–	–	–	–	−0.17213	0.035053
1,6-bis[4-(4-aminophenoxy)phenyl] diamantane	mRNA	ALG3	Expression	CellMiner	–	–	–	–	0.46380728	0.009709555	–	–
antineoplastic-131513	mRNA	ALG3	Expression	CellMiner	–	–	–	–	–	–	0.501695	0.0174
Benzethonium Chloride	mRNA	ALG3	Expression	CellMiner	–	–	–	–	–	–	0.408645	0.023312
indole-2,3-dione, 3-[(o-nitrophenyl)hydrazone]	mRNA	ALG3	Expression	CellMiner	–	–	–	–	–	–	−0.53322	0.002584
sb-590885-aad	mRNA	ALG3	Expression	CellMiner	–	–	–	–	0.4544494	0.007762472	0.431296	0.042454
sempervirine, nitrate, dihydrate	mRNA	ALG3	Expression	CellMiner	–	–	–	–	–	–	0.381832	0.034496
BX-912	mRNA	ALG3	Expression	GDSC	–	–	–	–	–	–	0.090154	0.010141
BX795	mRNA	ALG3	Expression	GDSC	–	–	–	–	–	–	0.085795	0.028814
CI-1040	mRNA	ALG3	Expression	GDSC	–	–	–	–	−0.12166483	0.001256026	−0.11084	0.003883
FH535	mRNA	ALG3	Expression	GDSC	–	–	–	–	–	–	−0.0935	0.03232
Genentech Cpd 10	mRNA	ALG3	Expression	GDSC	–	–	–	–	–	–	0.078334	0.029732
GSK429286A	mRNA	ALG3	Expression	GDSC	–	–	–	–	–	–	0.083435	0.017294
GW-2580	mRNA	ALG3	Expression	GDSC	–	–	–	–	–	–	0.07488	0.038217
Olaparib	mRNA	ALG3	Expression	GDSC	–	–	–	–	–	–	0.081806	0.036641
Selumetinib	mRNA	ALG3	Expression	GDSC	–	–	–	–	−0.1133474	0.001614548	−0.1253	0.00041
Tivozanib	mRNA	ALG3	Expression	GDSC	–	–	–	–	–	–	0.08174	0.019842
Trametinib	mRNA	ALG3	Expression	GDSC	–	–	–	–	−0.1248489	0.000340464	−0.12708	0.000241
UNC1215	mRNA	ALG3	Expression	GDSC	–	–	–	–	–	–	0.07031	0.047079
VX-11e	mRNA	ALG3	Expression	GDSC	–	–	–	–	−0.1036142	0.005889225	−0.10918	0.002903
